# Functionalized Optical Microcavities for Sensing Applications

**DOI:** 10.3390/nano15030206

**Published:** 2025-01-27

**Authors:** Evelyn Granizo, Pavel Samokhvalov, Igor Nabiev

**Affiliations:** 1 Laboratory of Optical Quantum Sensors, Life Improvement by Future Technologies (LIFT) Center, Skolkovo, 143025 Moscow, Russia; aleroman16@hotmail.com (E.G.); or p.samokhvalov@gmail.com (P.S.); 2 Laboratory of Nano-Bioengineering, National Research Nuclear University MEPhI (Moscow Engineering Physics Institute), 115409 Moscow, Russia; 3 Department of Clinical Immunology and Allergology, Institute of Molecular Medicine, Sechenov First Moscow State Medical University (Sechenov University), 119146 Moscow, Russia; 4 BioSpectroscopie Translationnelle (BioSpecT)—UR 7506, Université de Reims Champagne-Ardenne, 51100 Reims, France

**Keywords:** optical microcavities, functionalization, sensing applications

## Abstract

Functionalized optical microcavities constitute an emerging highly sensitive and highly selective sensing technology. By combining optical microcavities with novel materials, microcavity sensors offer exceptional precision, unlocking considerable potential for medical diagnostics, physical and chemical analyses, and environmental monitoring. The high capabilities of functionalized microcavities enable subwavelength light detection and manipulation, facilitating the precise detection of analytes. Furthermore, recent advancements in miniaturization have paved the way for their integration into portable platforms. For leveraging the potential of microcavity sensors, it is crucial to address challenges related to the need for increasing cost-effectiveness, enhancing selectivity and sensitivity, enabling real-time measurements, and improving fabrication techniques. New strategies include the use of advanced materials, the optimization of signal processing, hybrid design approaches, and the employment of artificial intelligence. This review outlines the key strategies toward enhancing the performance of optical microcavities, highlights their broad applicability across various fields, and discusses the challenges that should be overcome to unlock their full potential.

## 1. Introduction

Optical microcavities attract much interest in optical sensing due to their capacity for leveraging the fundamental properties of light and the resonant effects for detecting subtle changes in their surroundings. This makes them highly versatile and attractive for a broad range of analyses, including measurements of physical and chemical parameters (e.g., temperature, humidity, stress, radiation, and pH) and the detection of biological markers (e.g., enzymes, nucleic acids, and antibodies) [1,2,3,4].

Recent advances in optical microcavity technologies have enabled the development of sensors that are not only more sensitive but also more accurate in detecting specific targets in complex and noisy environments. These improvements are essential for applications where accuracy and reliability are crucial, such as cancer diagnosis. Due to enhanced sensor performance, including higher sensitivity and selectivity, optical microcavities can be used for precise and reliable measurements, ultimately advancing decision-making processes in critical applications.

Despite these advances, significant limitations remain. One key limitation is the challenge of maintaining high sensitivity and selectivity in diverse and dynamic environments, particularly if trace amounts of analytes are to be detected and if operation under real-time conditions is required. The need for scalability and cost-effective fabrication methods also requires further improvement to ensure the broader adoption of these technologies. Additionally, the integration of novel materials and hybrid configurations in order to expand sensing capabilities remains insufficient, leaving room for innovations in both design and implementation.

This review explores the latest developments in optical microcavity sensors and highlights how functionalization enhances their sensing capability. We provide an overview of the key materials used to improve sensor configurations and the various techniques employed to optimize the performance of optical microcavities used for sensing. We also discuss the challenges facing the further development and application of these technologies.

## 2. Functionalized Optical Microcavities

**Figure 1:**
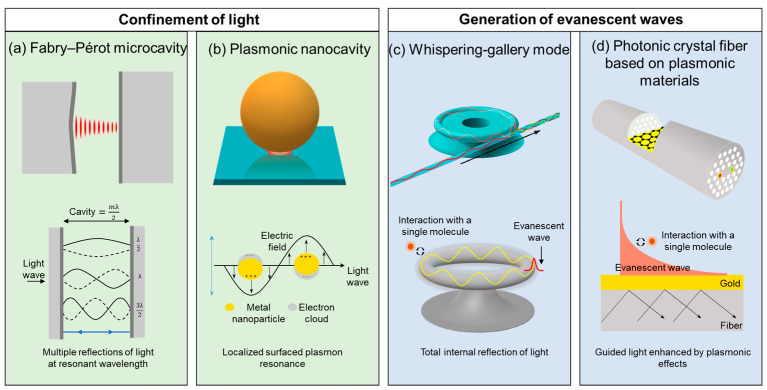
Schematics of the physical structure of various optical microcavities and the main physical principles used for sensing.

Optical microcavities, such as Fabry–Pérot (FP) microcavities, microcavities based on plasmonic materials (e.g., photonic crystal fiber (PCF) plasmonic sensors and plasmon nanocavities), and whispering-gallery mode (WGM) microcavities (e.g., microring resonators) are emerging as powerful tools in sensing technologies. Depending on their configuration, these microcavities can either concentrate light, as Fabry–Pérot microcavities and nanocavities do, or generate evanescent waves at the interface of dielectric and plasmonic materials, as PCF plasmonic sensors and WGM microcavities do (Figure 1).

Fabry–Pérot microcavities operate on the principle of multiple reflections between two parallel, highly reflective surfaces. Resonance occurs when the optical path length (i.e., the distance between the reflective surfaces) is an integer multiple of half-resonance wavelength, allowing specific wavelengths to be confined within the cavity (Figure 1a).

The strong confinement and enhancement of electromagnetic fields in plasmonic nanocavities arise from the collective oscillation of free electrons in the metal in response to incident light at specific frequencies [5]. These nanocavities generate “hot spots” with electric field intensities exceeding those of the incident light, enabling a significant enhancement of light–matter interactions. Specifically, the strong local field enhancement in plasmonic nanocavities, combined with their ability to interact with individual molecules, enable plasmonic nanocavity sensors to achieve exceptional sensitivity, which is crucial for single-molecule detection. When a molecule interacts with these nanocavities, the local field enhancement boosts the interaction between the molecule and the incoming light. This can lead to detectable changes in various characteristics, such as light scattering, light absorption, and fluorescence parameters (Figure 1b).

Whispering-gallery mode microcavity sensors employ specific wavelengths of light circulating within the microcavity, confined by total internal reflection and forming highly localized optical fields. Part of the confined light extends slightly outside as an evanescent field, which decays exponentially [6]. Interactions of the evanescent field with nearby analytes, including molecules and nanoparticles, perturb the WGM resonance, allowing the detection of the refractive index (RI) due to frequency shifts (Figure 1c).

Photonic crystal fiber plasmonic sensors employ guided light confined in a periodic dielectric structure and enhanced by plasmonic effects. At the interface between a metal and a dielectric, incident light excites collective oscillations of conduction electrons in the metal, creating an evanescent wave used for sensing [7] (Figure 1d).

The two main detection mechanisms used with functionalized optical microcavities are based on recording refractive index changes or fluorescence. The RI unit (RIU) is a metric for evaluating the performance of microcavities in RI sensing. It is defined as the smallest detectable change in the refractive index of an analyte [8]. Compared to other sensing systems, optical microcavities stand out for their superior performance due to two key factors: their capacity for confining light within small volumes and their high potential for localized and highly efficient chemical functionalization. This combination enables the sensitive and selective detection of physical, chemical, or biological changes in surrounding media.

Functionalized optical microcavities combine materials with different optical properties, such as high-reflectance materials (e.g., Bragg mirrors), plasmonic nanoparticles (e.g., gold and silver ones), and quantum dots (QDs). These materials augment the detection capability of the microcavity by enhancing light–matter interactions and boosting sensitivity and selectivity. Additional strategies include the use of novel materials and hybrid and other advanced designs, such as enhanced surface functionalization, and microfluidics [9]. Furthermore, employing artificial intelligence (AI) algorithms to filter noise and reduce false positives offers a path to even greater precision of detection. Despite the resultant advantages, such as rapid response and versatility, challenges still remain in terms of the ease of fabrication, long-term stability, and material compatibility.

One of the key approaches involving plasmonic materials employs the surface plasmon resonance (SPR) and localized surface plasmon resonance (LSPR) phenomena. These effects ensure better light confinement and are foundational for highly sensitive sensors that respond to changes in the refractive index caused by analyte binding. Recent advances in nanometer-precise lithography and colloidal synthesis have enabled the fine tunability of LSPR in the visible and near-infrared ranges, broadening the application scope of these sensors.

Polymers are also used in optical microcavities due to their flexibility, transparency, and biocompatibility [10]. The main developments in this area have focused on improving the material properties, fabrication techniques, and integration strategies, including block copolymer self-assembly and layer-by-layer assembly for precise control of the film thickness and composition [11]. Advanced techniques used with these materials include electrospinning in the case of ultrafine polymer fibers with a high surface-area-to-volume ratio and photopolymerization in the case of submicron-resolution 3D structures. They reduce manufacturing time and support complex designs [12].

Two-dimensional (2D) materials, such as graphene and transition metal dichalcogenides, show great promise for sensor applications due to their ultrathin structures and exceptional sensitivity. These materials reduce losses, improve biocompatibility, and ensure molecular specificity [13], which makes them ideal for applications in the early diagnosis of a number of diseases, including cancer [14]. For example, graphene stands out for its exceptional properties, including a high electrical conductivity, a large surface area, chemical inertness, and thickness (about 0.3 nm [15]), which make it a prime candidate for sensor applications [16].

Quantum dots, semiconductor nanocrystals ranging from 1 to 12 nm in diameter, have unique optical and electronic properties, including high quantum yields, long fluorescence lifetimes, large extinction coefficients, narrow emission spectra, and high photostability. These properties can be tuned by adjusting their size [17], composition, or surface ligands [18]. Their surface chemistry can be modified to improve the sensor specificity, enabling their integration with various detection methods, such as fluorescence, electrochemistry, and Raman scattering measurements. By combining QDs with other nanomaterials, e.g., molecularly imprinted polymers and noble metal nanoparticles, sensor performance can be significantly improved. These hybrid systems are particularly effective in detecting complex analytes, including antibiotics and metal ions, by leveraging both the optical properties of QDs and the functionalities of the other materials [19]. However, there still remain challenges related to optimizing real-world applications, particularly, reducing the QD toxicity.

In biosensing applications of sensors based on optical microcavities, bio-recognition elements play a critical role in enabling high sensitivity and specificity. When the target analyte is recognized and bound by the sensing element of the sensor through specific molecular interactions, its physical properties, such as the refractive index, are changed. Bio-recognition elements include antibodies, enzymes, nucleic acids, aptamers, phages, peptides, lectins, and molecularly imprinted polymers, all of which selectively interact with the target molecules [20,21,22].

The further improvement of functionalized optical microcavities is essential for advancing various sensing applications. Current efforts are focused on enhancing sensor performance for detecting smaller analyte quantities, achieving faster real-time responses, and enhancing selectivity in complex environments. Future developments in functionalized microcavity designs are aimed at increasing the sensitivity to the degree where single molecules can be detected and expanding the range of applications of the microcavities.

## 3. Fabry–Pérot Microcavities

Fabry–Pérot microcavities are optical resonators consisting of two reflective surfaces, such as metal layers or Bragg mirrors (one-dimensional photonic crystals), separated by a central cavity. This configuration enables the confinement of light inside the cavity, facilitating precise optical interactions and high resonance quality. Common materials used for the fabrication of FP microcavities include metals, such as gold and silver, known for their excellent reflective properties; dielectric materials, such as TiO_2_, Ta_2_O_5_, ZnS, GaAs, SiO_2_, and MgF_2_; and semiconductors, such as Si, Ge, and GaN. More recent designs use novel combinations, e.g., Ag/Si [23] and Si/Si_3_N_4_ [24], which substantially improve the performance of these microcavities by ensuring the optimal photonic bandgap structure and a superior refractive index contrast.

Fabry–Pérot microcavities offer a powerful research platform that enable precise light–matter coupling, offering high-quality light confinement and strong resonance effects. These properties make them effective for detecting refractive index changes due to environmental factors, with applications in biology, chemistry, and environmental monitoring, such as gas detection [25,26], food safety assessment and early cancer biomarker detection, where timely intervention can significantly improve treatment outcomes [27]. When the physical properties of FP microcavities are modified, they become exceptionally sensitive tools for detecting variations in humidity, pressure, temperature [28], and ionizing radiation [29].

Obtaining SPR in FP microcavities further sharpens the resonance peaks (i.e., narrows the linewidths), which decreases the detection threshold. At the same time, LSPR provides a high spatial resolution due to localized field enhancement facilitated by metal nanoparticles. Combining these mechanisms in FP microcavities amplifies the resonance sensitivity and tailors the optical responses, thus enhancing the sensing capability. Some innovative designs use the metal/cavity/multilayer porous TiO_2_ photonic crystal structure to employ Tamm plasmon resonance for detecting traces of heavy metals [30]. Tamm plasmon resonance refers to the confinement of electromagnetic waves at the interface of a metal and a periodic dielectric mirror, resulting from the coupling of light with surface electron oscillations. It enables localized modes for applications in sensing [31]. FP microcavities can support Tamm plasmon resonance if properly configured, which results in hybrid Tamm plasmon–cavity modes. These configurations have the potential for higher sensitivity [32] and detection accuracy [33]. Nanoparticles embedded in the layers further enhance the detection capability by boosting refractive index contrasts [34,35].

**Figure 2:**
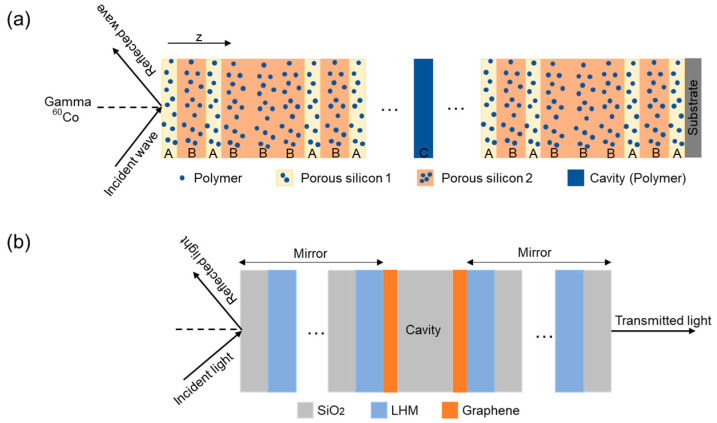
(**a**) Schematics of an optical microcavity used for gamma radiation detection. The microcavity is made of porous silicon layers with a high refractive index contrast, doped with a polyvinyl alcohol, carbol fuchsin, and crystal violet (Layers A and B). The cavity itself is formed from the same polymer composition (Layer C). (**b**) Schematics of an optical microcavity made of metamaterial (LHM), SiO_2_, and graphene sheets at the interface between cavity and mirror.

Polymers and inorganic materials are also employed in FP microcavity designs to expand their functional capabilities. For example, simulation predicts an impressive sensitivity of gamma radiation sensors based on porous silicon doped with polymers, which makes these configurations valuable for medical applications [29] (Figure 2a). The sensor incorporates porous silicon layers with a high refractive index contrast, doped with a polymer of polyvinyl alcohol, carbol fuchsin, and crystal violet (Layers A and B in Figure 2a). The cavity itself is formed from the same polymer composition (Layer C in Figure 2a). The results show a sensitivity to gamma radiation of 0.265 nm/Gy and a QF of 12,701. The incorporation of QDs into FP microcavities has proven versatile, enabling applications where single-photon sources [36] are required. In sensing, to further improve the sensitivity of porous silicon microcavity biosensors, water-soluble CdSe/ZnS QDs have been proposed for amplifying the refractive index signal as a consequence of the high refractive index of QDs [37]. This further highlights the potential of QDs for real-time, portable monitoring and low detection limit applications [38].

Two-dimensional materials, including graphene and WS_2_ monolayers, have been explored in terms of improving the optical properties of FP microcavities [39]. Innovative designs, such as graphene-embedded defect one-dimensional photonic crystals, have enabled the real-time identification of various cancer cell types, including basal, cervical, and breast cancer cells [40]. In addition, coupling FP microcavities with metamaterials, e.g., MXenes, at the interface between the cavity and the mirror and the optimization of the cavity parameters ensure sharp, distinguishable resonance peaks and enhanced sensor performance [41]. Apart from refractive index sensing, FP microcavities offer tunable absorption properties, which can be varied by adjusting their design and material composition. Incorporating graphene sheets at the interface between the cavity and the mirror (Figure 2b) and using metamaterials with negative permittivity and permeability have been shown to enhance energy absorption, making them valuable for photodetector systems. Metamaterials, such as left-handed materials (LHMs), enhance electromagnetic wave absorption due to their negative refractive index increasing the path length and absorption efficiency [42].

Similarly, some configurations of FP microcavities based on dielectric (MgF_2_ or ZnSe) Bragg mirrors and a graphene monolayer at the interface between the cavity and the mirror exhibit exceptional sensitivity, signal-to-noise ratio, and resolution metrics for applications in cancer cell detection. The sensors based on these microcavities have a sensitivity as high as 290 nm/RIU and a quality factor of 2270.74 [40]. These advancements meet the growing demand for miniaturized biosensors, particularly those designed for point-of-care cancer detection.

Metal–organic frameworks have also been integrated into FP microcavities. In this case, the sensing benefits from their high specific surface area and customizable functionalities [43]. These properties of metal–organic frameworks enhance the microcavity interactions with analytes, and the functional groups can target specific molecules. FP microcavities based on metal–organic frameworks are used, e.g., in colorimetric sensing, where variations in the thickness and optical density of the FP film provide precise detection. Another example is humidity sensing [44,45]. Thus, the use of metal–organic frameworks further broadens the scope of applications of FP microcavities.

The versatility of FP microcavities in biosensing is augmented through the functionalization of the sensing area with receptor molecules. When target biomolecules bind to these receptors, a measurable shift in the resonant response occurs, facilitating the detection of a wide range of biomarkers [46,47].

A promising novel sensor not requiring probe pre-modification is an optofluidic microbubble placed inside an FP microcavity. This design combines a high-quality factor (~10^5^), small mode volume (approximately one-sixth of the mode volume of the original FP microcavity), and high robustness (i.e., good tolerance to non-parallelism (5°) and cavity length changes). These features enable an ultrahigh refractive index sensitivity (679 nm/RIU) and an ultrafine refractive index resolution (~10^−7^ RIU at 950 nm), representing a significant improvement over the original FP microcavity, which had a refractive index resolution of ~10^−4^ RIU. This configuration provides a low detection limit and a wide detection range for biomolecules, e.g., several femtograms per milliliter for IgG and less than a picogram per milliliter for human serum albumin [2]. Another important example is a hybrid optofluidic microcavity composed of a microsphere and FP microcavities, which is characterized by exceptionally low effective mode volumes (0.3–5.1 μm^3^) and high quality factors (from 1 × 10^4^ to 5 × 10^4^). This hybrid design leverages the advantages of both components: the microsphere confines light to a small mode radius (0.26–0.9 μm) and mode volume, provides beam focusing due to the lensing effect, and mitigates geometrical losses by refracting or reflecting misaligned light [48]. In this configuration, the microsphere serves as both a waveguide and a lens, enhancing the overall performance of the system. In addition, a convex microlens array structure inside the FP cavity has been proposed, which ensures a 5.6-fold increase in the quality factor compared to the original FP cavity [49].

Porous silicon microcavities, in particular, have gained much attention for their low cost, easy fabrication, and fine control over the refractive index of the material. Recent developments include the use of aptamers for protein biomarker detection, enhanced by the addition of 3D-printed microfluidic systems with micromixer architectures [50]. These innovations have improved sensor performance, ensuring detection limits as low as 50 nM. The integration of diverse materials and mechanisms in FP microcavities further extends their research, technological, and medical applications, reinforcing the potential of FP microcavities in advanced sensing and diagnostic technologies.

Furthermore, FP microcavities are integral to compact and stable spectrometer designs. SERS-active materials based on FP microcavities have proven to be two orders of magnitude more sensitive than conventional ones based on silicon substrates [51]. By addressing challenges related to achieving a high spectral resolution and overcoming manufacturing constraints, spectral reconstruction algorithms have been developed. These innovations significantly enhance the resolution and functionality of spectrometers, making them suitable for various high-precision applications.

Recent advancements have also extended the capability of FP microcavity sensing to the degree required for terahertz frequency-domain spectroscopy [52], further broadening their application scope. The versatility of FP microcavities makes them all the more important as promising tools across a wide range of applications, driving innovation and discovery in science and technology.

## 4. Plasmonic Microcavity-Based Sensors

Plasmonic materials, particularly gold and silver, are promising for sensing applications due to their capacity for supporting SPR and LSPR, enabling enhanced optical sensitivity and precision. SPR occurs in thin metal films when the frequency of the evanescent wave matches that of the surface plasmons, enabling broadband light confinement [53]. In contrast, LSPR is induced by electron oscillations confined to metal nanoparticles and is highly sensitive to the size, shape, and composition of the particles, as well as the refractive index of the surrounding medium. Nanoparticles, in particular, offer unique advantages at the molecular level because their small size minimizes bulk effects typical of films. This means that only the bound molecule contributes to the LSPR signal, which makes it possible to detect single molecules [54,55]. This makes LSPR ideal for detecting analytes in a subwavelength-scale volume. The combination of the advantages offered by the SPR and LSPR effects and the use of 2D materials in plasmonic cavity sensors has been shown to improve a number of performance parameters, such as the sensitivity, limit of detection (LOD), and detection range [56,57]. This combination has been extensively studied in terms of applications in gas sensing and biosensing, including cancer biomarker detection [58]. Plasmonic integrated systems are also used for chemical sensing, temperature monitoring [59], humidity and pressure measurements [60], as well as food safety and environmental monitoring [61].

These plasmonic materials have been integrated into different microcavity designs, including plasmonic PCF sensors. PCFs based on plasmonic materials are optical fibers with air channels forming ordered arrays that efficiently guide and confine light by reflecting it internally. These microcavities, with enhanced light–matter interactions, constitute a highly sensitive alternative to conventional fibers. Geometric parameters, such as the channel size, spacing, and the thickness of the plasmonic metal film, significantly affect the wavelength sensitivity.

The sensitivity of PCF SPR sensors is largely determined by the plasmonic material used. Typically, this is silver, gold, aluminum, copper, bismuth, or palladium, with gold standing out for its high chemical stability and a large change in the resonant peak upon interaction with the analyte [60]. The innovative designs of plasmonic PCFs, elaborated with the intense use of computational methods, ensure high spectral sensitivity and resolution [62,63]. For example, D-type fiber-optic sensors are easy to fabricate and allow for the integration of gold films near the core to obtain strong evanescent waves that interact effectively with the surrounding medium, which enhances the biosensor sensitivity [64].

**Figure 3:**
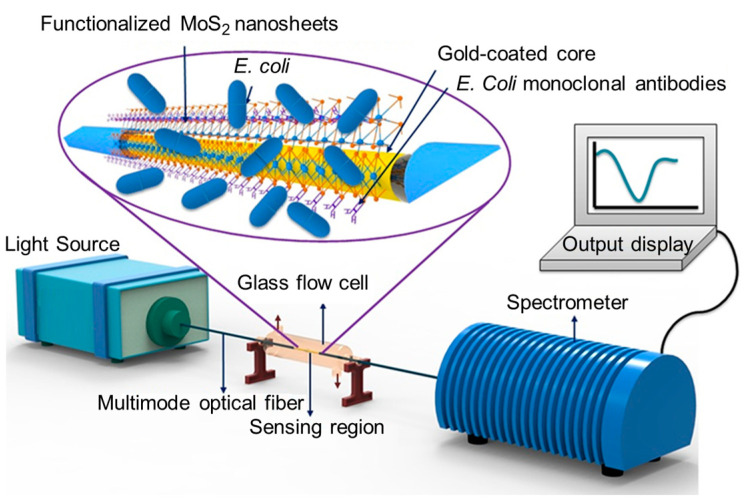
Schematic representation of the experimental setup of the developed fiber-optic SPR immunosensor for the detection of *E. coli.* Modified with permission from Kaushik et al. (2019) [63], published by Elsevier, Amsterdam, The Netherlands.

As noted above, SPR sensor performance can be enhanced by incorporating 2D materials, such as graphene, which strongly interact with biomolecules to significantly increase the sensitivity. These SPR sensors detect subtle variations in the refractive index. For instance, a maximum amplitude sensitivity of 14,847.03 RIU^−1^ and an average wavelength sensitivity of 2000 nm/RIU have been demonstrated for graphene-based SPR sensors [65]. The addition of graphene oxide to gold nanoparticles improves biomolecule adsorption, reducing the LOD to a nanogram scale [66]. Graphene combined with other materials, e.g., magnesium fluoride (MgF_2_), has been used to coat PCF SPR sensors. MgF_2_ is an ideal material for coatings due to its capacity for supporting a wide wavelength range, from ultraviolet to infrared, and facilitating the excitation of surface plasmon waves, thereby enhancing sensor performance. By adding a MgF_2_ layer, the average wavelength sensitivity and amplitude sensitivity have been increased from 2500 nm/RIU^−1^ to 4000 nm/RIU^−1^ and from 12,155.2 RIU^−1^ to 16,905.15 RIU^−1^, respectively. This sensor has been proposed for detecting analytes in aqueous solutions [67]. Molybdenum disulfide (MoS_2_) nanosheets are another example of a 2D material enhancing the SPR sensor sensitivity [68]. The MoS_2_ nanosheets are anchored to the gold layer surface, their function being to promote the immobilization of monoclonal antibodies through hydrophobic interactions. This strategy has been used for the quantitative analysis of *E. coli* with a sensitivity of 3135 nm/RIU (Figure 3).

Another approach to enhancing the performance of plasmonic sensors is to protect the plasmon layer from oxidation. For example, the coating of the silver layer with phosphorene has been reported to increase the wavelength sensitivity to 1800 nm/RIU within a refractive index range of 1.39–1.45 and the maximum amplitude sensitivity to 268 RIU^−1^, with an analyte refractive index of 1.40 [69]. Other materials, such as titanium dioxide (TiO_2_), are also employed to protect silver films from oxidation and enhance the SPR effect [70].

The combination of various prospective materials discussed above in a single-sensor architecture leads to additional improvements of plasmonic sensors. For instance, combining gold as the primary plasmonic layer with graphene and Ti_3_C_2_Tx-MXene increases the sensitivity and stability, facilitating the early detection of cancer biomarkers at small concentrations [71]. Carbon nanotubes and graphene oxide amplify signals, improve sensitivity, and provide a high chemical stability in fiber optic-based biosensors, with carbon nanotubes promoting molecularly imprinted polymer formation and graphene oxide enhancing the interaction between the SPR and the molecules [72,73]. QDs are incorporated into functionalized sensing systems to improve the detection of antibiotics, biomarkers, and metal ions [19]. For example, CsPbBr_3_ QDs enhance the energy transfer from the core mode of PCFs to the SPR mode of the metal, boosting the sensing performance while avoiding a narrow full width at half maximum [74].

A recent breakthrough is an immunosensor based on a fiber-optic Fabry–Pérot interferometer for single-molecule detection that combines AuNP-embedded graphene oxide with a D-shaped optic fiber to address the challenges of the low spectral contrast and limited area of contact between light and material. The sensitivity and selectivity of the sensor are improved due to the localized confinement of the electromagnetic field of gold nanoparticles, ensuring an ultrahigh refractive index sensitivity of 583,000 nm/RIU and an LOD of 17.1 ag/mL in the case of progastrin-releasing peptide detection [75]. Similarly, combining ZnO nanowires and WS_2_ nanosheets provides a wide surface area for functionalization and antibody/nanoparticle immobilization in plasmon wave-based fiber sensors, providing a sensitivity of detection of 1.32 ng/mL and an LOD of 84 pg/mL for an alpha-fetoprotein solution at concentrations below 1000 ng/mL [76,77]. GO-enhanced sensors have been found to be 2.5 times more sensitive than gold-coated fiber-optic sensors [78].

Despite these advances, challenges remain in scaling up the fabrication of PCF sensors for commercial use due to the high production costs and complexity. Researchers are continually exploring ways to simplify the sensor design in order to facilitate miniaturization and increase cost-effectiveness while ensuring a high sensitivity for leveraging the potential of plasmonic sensors in medical diagnosis, environmental monitoring, and other fields. Recent trends in these developments include the use of microstructured fibers, the optimization of the sensor geometry, and the fabrication of hybrid systems.

Combining microcavity and plasmonic effects has been demonstrated to ensure ultrasensitive detection. For example, a hybrid microcavity-based SERS sensor provides detection limits of 3.16 pg/mL for cardiac troponin I and 4.27 pg/mL for creatine kinase MB, outperforming the traditional fluorescence and ELISA techniques [79]. Recent SERS developments in microfluidic systems have enabled femtogram-level detection for the biomarker of Alzheimer’s disease and Epstein–Barr virus [80,81]. SERS enables the ultrasensitive detection of analytes down to single molecules, using a metal–dielectric nanocavity engineered from the SARS-CoV-2 RBD protein and silver. By enhancing the quality factor of the cavity with a silver shell, a sub-femtogram sensitivity for viral antigen detection is achieved without the use of Raman reporter molecules. This label-free method allows high-performance optical detection and conformational analysis of viral proteins at physiologically relevant levels [82].

Surface-enhanced Raman scattering analysis is a powerful analytical technique offering remarkable sensitivity that allows single-molecule detection. However, it has substantial limitations. One key challenge is the need for reproducibility of the SERS signals, because the technique relies on the quality and uniformity of nanostructured substrates, which can vary during fabrication. Commonly used substrates, such as silver, are prone to oxidation and degradation, which reduce the performance over time. SERS also struggles with complex sample matrices, where nonspecific adsorption, interfering signals, or matrix effects may hinder accurate detection. Additionally, some types of molecules with weak Raman activity or without strong affinity to SERS-active surfaces (e.g., nonpolar/hydrophobic molecules) are difficult to detect. The performance also depends on environmental factors, such as pH, temperature, and ionic strength. The high cost of SERS analysis and the complexity of substrate preparation also limit its scalability for routine applications.

The ongoing development of novel designs increases the versatility of plasmonic microcavity-based sensors, enabling, e.g., single-molecule detection. However, further research is needed to address manufacturing challenges and improve scalability.

## 5. Whispering-Gallery Mode Microcavities

Whispering-gallery mode microcavities, such as microdisks, microtoroids, and microspheres, are distinguished by their high quality factors, high sensitivity, and long photon confinement times. These properties make them ideal for advanced light–matter interaction studies and a wide range of sensing applications, such as the detection of biomolecules and chemical substances (including gasses) and temperature, pressure, humidity, and strain sensing. These innovations demonstrate the potential of WGM microcavities in revolutionizing precision sensing across various research and industrial applications.

**Figure 4:**
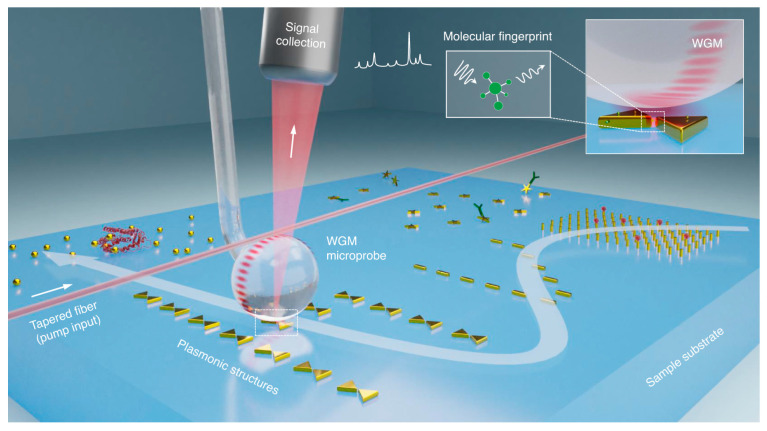
Schematic of the combination of whispering-gallery modes and nanoplasmonics for enhancing Raman spectroscopy. Reproduced from Mao et al. (2023) [79], published by Springer Nature under a Creative Commons Attribution 4.0 International License http://creativecommons.org/licenses/by/4.0/.

When combined with plasmonic nanoparticles or thin metal layers, WGM resonators significantly enhance light–matter coupling. This configuration has proven useful in detecting ultrasmall virus particles, such as the MS2 RNA virus, even at very low concentrations [83]. Plasmonic-enhanced WGM systems are particularly valuable in cavity quantum electrodynamics, single-photon sources, and sensing because they enable signal enhancement beyond the detection limits of conventional biosensors. The combination of whispering-gallery modes and nanoplasmonics can ensure an almost two times more intense Raman spectroscopy signal for sensitive molecular fingerprinting compared with the traditional purely plasmonic approach [84] (Figure 4). Moreover, nanoparticles deposited on the surface of a WGM microcavity can enhance Rayleigh scattering, facilitating effective WGM coupling [85].

Whispering-gallery mode microcavities are categorized into passive and active types. Passive WGM microcavities are generated in an undoped dielectric material and do not emit light by themselves. They are widely used in high-sensitivity sensing due to their high quality factors. Active WGM microcavities, on the other hand, occur in gain materials, such as dyes and QD-doped microcavities, where light amplification via stimulated emission enables, e.g., fluorescence-based sensing [86].

Originally fabricated from silica, WGM microcavities have also been made of polymers in recent years. Polymers offer significant advantages, such as low cost, adaptability, and compatibility with diverse fabrication techniques [87]. Their inherent elasticity makes them suitable for strain or deformation sensing, and their tunable refractive indices enable the integration of bioactive molecules, including antibodies, enzymes, and DNAs. Such functionalized WGM sensors detect changes in refractive index upon target molecule binding, producing strong optical signals. For example, microring resonators fabricated by femtosecond laser two-photon photopolymerization (TPP) have a temperature sensitivity of 9.51 × 10^−2^ nm/°C and a refractive index sensitivity of 12.50 nm/RIU, which shows their potential for precise sensing applications [88]. However, photopolymerization faces some challenges, such as the need for specific light-sensitive monomers and restricted light penetration depths, which may limit its applicability in some sensor designs. Optimizing the designs, such as incorporating tapered waveguides into polymer ring resonators or increasing the undercut size in WGM microdisks, enhances the sensitivity and decreases the detection threshold. The use of biodegradable materials for healthcare applications, particularly in transient sensing devices for personalized diagnosis, represents a growing area of research. The use of polymers further advances the optical sensor technology, with ongoing efforts to address challenges related to stability and performance under varying environmental conditions. Innovations in fabrication methods, such as drop-on-demand inkjet printing for optical gas sensors employing polymer WGM microcavities, have enabled the integration of multiple sensing materials on a single microchip. These systems have quality factors exceeding 10^6^ [89].

The incorporation of 2D materials represents another feasible way to improve the performance of WGM-based optical devices and promote the development of novel photonic and optoelectronic devices employing enhanced light–matter interaction [90]. Functionalizing over-modal microcavities with a single graphene layer has been used to engineer microcomb sensors with high chemical selectivity and sensitivity [91]. Similarly, MXene nanosheets, such as Nb_2_C ones, have been integrated into WGM microcavities to enhance electrochemiluminescence (ECL), enabling the sensitive detection of miRNA in triple-negative breast cancer. Here, Nb_2_C nanosheets are MXene-derived luminescent materials and SiO_2_-optical microspheres are assembled on the electrode surface to form a WGM-optical microcavity that localizes photons and amplifies the ECL signal [92]. In addition, the possibility of multimode sensing using WGM microcavities has been demonstrated, which further enhances the resolution and dynamic range of sensing [93].

These advances, coupled with continuous innovations in materials, optimized geometries, and fabrication techniques, further expand their applicability and improve their performance, making them indispensable tools in next-generation sensing systems.

## 6. The Advantages and Drawbacks of Different Microcavity Designs for Sensing Applications

Fabry–Pérot microcavities have a larger functional surface area compared to other optical microcavity designs, enabling enhanced sensor performance upon functionalization with specific receptors or nanomaterials. However, whereas they have a higher quality factor than plasmonic cavities, their light confinement volume is significantly larger than that of nanoparticle-based microcavities, precluding the detection of single molecules. This limitation could be overcome by making the reflective surfaces convex or concave or by incorporating an array of convex microlenses into the FP microcavity [49]. Additionally, spectral tuning using (electro)mechanical positioners has been explored as a method for precisely adjusting the spacing between the mirrors [36,94]. Some materials, such as porous silicon, offer tunability to meet various detection requirements and are cost-effective and simple to manufacture. However, the scalability of their production remains a problem because they are sensitive to the manufacturing conditions, such as the resistivity of silicon wafers, temperature, and etching solutions. Further research is needed to optimize the microcavity designs, refine the adjustment of the parameters, and enhance long-term stability.

In contrast, SPR-based microcavities have small mode volumes, enabling single-molecule detection. Although their quality factors are often low because of high losses in the metal, the combination with 2D nanomaterials increases their capacity for functionalization and biocompatibility. These advantages are particularly obvious in the case of PCFs, where electric field confinement is further optimized. In particular, D-shaped fibers are easy to fabricate and functionalize while retaining a high sensitivity. Novel designs of these systems offer improved adaptability for a variety of detection applications. Nevertheless, challenges persist in scaling up their production because the precise and controlled manufacturing of each component is required.

Regarding WGM microcavities, despite their exceptional sensitivity and resolution, several limitations hinder their broader application. Tracking specific modes confines sensing performance to a single data channel, complicating multimodal data collection. The limited dynamic range of WGM sensors poses an issue because mode shifts beyond the laser scanning range disrupt measurements; on the other hand, extending the range often sacrifices resolution. Furthermore, noise interference reduces the accuracy of detecting subtle changes, whereas the necessity of reliably tracking the same mode interferes with reproducibility and standardization. Addressing these issues is critical for expanding the applicability of WGM sensors [93].

In conclusion, while Fabry–Pérot, SPR, and WGM microcavities offer unique advantages in sensor performance, they also face challenges that limit their broader application. Fabry–Pérot microcavities have large functional surfaces but do not allow single-molecule detection due to their larger light confinement volume. SPR microcavities excel at single-molecule detection but face issues with low quality factors and scalability. Specifically, SERS techniques have been optimized for analyte detection at ultralow concentrations, even in complex samples, through electromagnetic field confinement. WGM microcavities provide high sensitivity and resolution, but they are limited by mode tracking, dynamic range, and noise interference. Further research is needed to address these limitations and improve the scalability, sensitivity, and reliability of these technologies for more diverse applications.

## 7. Prospects

Functionalized microcavities have proven remarkably versatile in biosensing, environmental monitoring, chemical and physical sensing, and other applications. The use of novel materials and approaches, including artificial intelligence [95], has significantly enhanced sensor performance, increasing the sensitivity, selectivity, and the capability for detecting low analyte concentrations in complex environments. This is particularly critical for cancer diagnosis [40]. The combination of different microcavity configurations and techniques, such as SERS-based sensing integrated with Fabry–Pérot, plasmonic microcavity sensing or WGM, within a single sensor helps overcome many limitations, leading to the synergistic enhancement of performance.

Recent advances in fabrication methods and technologies allow better control over the sizes and geometric parameters, which are crucial for sensor designs where performance is highly sensitive to dimensional accuracy. Despite these improvements, even more precise methods are required to achieve consistent performance. Miniaturization and combination with microfluidics are essential for adapting sensors to real-world environments, enabling lab-on-chip configurations suitable for commercial applications. However, practical implementation remains a challenge requiring new solutions for cost reduction, scalability, and user-friendliness.

Whispering-gallery mode sensors have already been demonstrated to have high potential in biological detection. However, their extreme sensitivity to environmental factors necessitates strategies for noise reduction, such as advanced data processing algorithms. Simplifications of design have resulted in fiberless systems [96], with polymers emerging as viable materials for cost-effective manufacturing. Miniaturized spectrometers are being developed to further enhance the portability and applicability of the sensors. In the case of FP cavities, optimizing fabrication processes to improve the quality factor and reproducibility remains an essential goal. Similarly, PCFs require more precise manufacturing to meet the optical requirements necessary for homogeneity and good performance in assembling nanomaterials.

Artificial intelligence plays a transformative role in improving sensor performance, enabling the processing of complex signals and the recognition of the patterns that would otherwise be undetectable. By combining the advantages of different configurations, e.g., balancing the optimal confinement volumes and quality factors, researchers are pushing the boundaries of multiplexed and multimodal detection, providing a wider range of analyte detection capabilities in the same device. For example, the integration of machine learning has been used for predicting the resonant peak wavelength of more complex systems, such as 2D PC biosensors used for cancer diagnosis. The use of machine learning models includes repeating training, testing, and optimization of the resonant wavelength with dependent and independent features of a 2D PC biosensor system [97]. In addition, machine learning has been used to predict reflectance, which can be used to optimize designs and reduce the simulation time [98]. The use of neural networks also contributes to enhancing the accuracy and efficiency of sensors, potentially revolutionizing hybrid optical–digital systems [99,100]. Hybrid sensor technologies integrating microfluidics [9], artificial intelligence, and the Internet of Things pave the way for next-generation lab-on-chip devices, tattoo-based sensors [101], and other groundbreaking innovations.

Spectral resolution is another key characteristic in sensing performance. Sensitivity alone does not ensure good performance if the system cannot detect small spectral shifts due to noise. Recent developments in multimode optical microcavity sensors have enabled multiplexed detection by using several resonant modes simultaneously, effectively acting as multiple sensors within a single device [102]. Here, machine learning-based multimode optical microcavity sensors employ algorithms to analyze the multimode shift data of the sensors and enhance the system performance by enabling efficient data fusion and pattern recognition. This approach reduces human error and accelerates analysis.

Efforts toward miniaturizing sensor devices, such as computational spectrometers with spectral encoding and reconstruction algorithms [103], will enhance their portability and accessibility. Additionally, the development of nontoxic, biodegradable materials will support sustainability and broader adoption across diverse applications. Future research should also focus on multichannel detection, hybrid sensors, and balance between performance parameters without sacrificing accuracy, with robust, scalable, and user-friendly sensor systems as the ultimate goal.

## 8. Summary and Outlook

Optical microcavities have been extensively explored in terms of their possible sensing applications in various fields, including the measurement of physical and chemical parameters and the detection of biological markers. Researchers have explored a variety of advanced materials, designs, and fabrication methods to improve the microcavity performance, with an ultimate goal to revolutionize sensing technologies. These advancements leverage the unique properties of optical microcavities, including various possibilities for functionalization, efficient electric field confinement, and strong interactions with analytes, enabling the development of highly sensitive and efficient sensors. Optical microcavity sensors integrated with nanomaterials offer exceptional sensitivity, resolution, and selectivity, enabling the detection of analytes at very low concentrations. Plasmonic materials, including metal films and metal nanoparticles, play a crucial role in improving sensor performance. SPR enables broadband light confinement and a larger surface area for functionalization, whereas the LSPR effect offers unique advantages at the molecular level due to the light confinement in small volumes, which further boosts the sensing capability, even to the degree of single-molecule detection. To increase specificity, optical microcavities coated with engineered nanomaterials can be functionalized with bio-recognition elements such as antibodies against the target analytes or markers. Other materials, such as 2D materials, metamaterials, polymers, and nanocrystals (QDs), are driving advancements in designing highly sensitive, flexible devices. Combining optical microcavities with microfluidics offers compact, effective solutions for bioanalytical applications, enhancing both performance and usability. The integration of machine learning algorithms allows more accurate data interpretation, making the devices more versatile and efficient by overcoming the limitations of traditional methods. Future trends in the development of these technologies are expected to focus on improving performance and scalability and facilitating their integration into multifunctional systems. Although functionalized optical microcavities have already demonstrated remarkable sensitivity, further efforts are called for to achieve miniaturization, sufficient cost-effectiveness, and applicability to real-time monitoring, and to ultimately expand their practical use in various fields.
